# Efficient CRISPR/Cas9‐based plant genomic fragment deletions by microhomology‐mediated end joining

**DOI:** 10.1111/pbi.13390

**Published:** 2020-05-22

**Authors:** Jiantao Tan, Yanchang Zhao, Bin Wang, Yu Hao, Yaxi Wang, Yangyang Li, Wanni Luo, Wubei Zong, Gousi Li, Shuifu Chen, Kun Ma, Xianrong Xie, Letian Chen, Yao‐Guang Liu, Qinlong Zhu

**Affiliations:** ^1^ State Key Laboratory for Conservation and Utilization of Subtropical Agro‐Bioresources Guangzhou China; ^2^ Guangdong Laboratory for Lingnan Modern Agriculture Guangzhou China; ^3^ College of Life Sciences South China Agricultural University Guangzhou China; ^4^ Chongqing Engineering Research Center for Floriculture College of Horticulture and Landscape Southwest University Chongqing China

**Keywords:** MMEJ, NHEJ, CRISPR/Cas9, genome editing, genomic fragment deletion

Clustered regularly interspaced short palindromic repeats (CRISPR)‐associated protein (Cas) systems produce double‐strand breaks (DSBs) in targeted sites and generally cause insertion/deletions of one or several bases by non‐homologous end joining (NHEJ)‐mediated DNA repair. However, many edited cell lines with small insertions/deletions produce abnormal transcripts or proteins causing unexpected effects that complicate functional analysis (Tuladhar *et al.*, 2019). Therefore, the ability to delete larger genomic fragments would have advantage for making true null alleles of coding and non‐coding genes. The NHEJ pathway also produces fragment deletion with low efficiency (Pathak *et al.*, 2019). Microhomology‐mediated end joining (MMEJ) is another DNA repair mechanism based on recombination between microhomologous sequences (MHSs) of 4–25 bp located near DSBs (Decottignies, 2007). Studies in model organisms including plants showed that MMEJ usually results in deletions of sequences between pairs of MHSs near DSBs generated by genome editing (Pathak *et al.*, 2019; Shen *et al.*, 2017). The MMEJ approach has been explored for deletion or insertion of genomic fragments in animals and microorganisms (Bae *et al.*, 2014; Nakade *et al.*, 2014; Zhang *et al.*, 2016), but this type of application as well as systematic investigation of the frequency of MMEJ events in plant genome editing is lacking.

In addition, generation of fragment deletions could remove antibiotic or herbicide selectable marker genes from transgenic lines for minimizing public and regulatory concerns. Several strategies for obtaining marker‐free transgenic plants have been reported (Perez and Angenon, 2013), but these methods require the use of a marker‐free system for plant transformation. Therefore, a high‐efficiency method for removing target DNA fragments in plants is needed.

Here, we analysed the efficiency of CRISPR/Cas9‐targeted deletion of genomic fragments mediated by MMEJ in rice (*Oryza sativa* L.) and demonstrated this approach as an effective tool for deleting marker genes from transgenic plants. Using the CRISPR/Cas9 multiplex genome editing system for plants (Ma *et al.*, 2015), our laboratory produced hundreds of rice mutant lines. A proportion of these lines were edited at two or more sites within the target genes. Although we did not consider MHSs for MMEJ in our initial experimental design, we selected 48 edited populations (each representing a group of T_0_ plants generated with the same CRISPR/Cas9 construct) that had fragment deletions between the target sites, and determined whether some deletion events were mediated by MMEJ (two MHS sites recombined as one in the deletion junctions) or by NHEJ (no MHSs in the deletion junctions). Of these edited populations, 24 had MMEJ‐mediated fragment deletions ranging from 28 to 20 667 bp with an average size of 1653 bp. The average fragment deletion efficiency was 18.9% (Figure 1a). Moreover, 43 edited populations had NHEJ‐mediated deletions ranging from 25 to 21 000 bp with an average size of 948 bp. Thus, if the design of editing targets does not consider facilitating MMEJ, the frequencies of fragment deletions induced by MMEJ and NHEJ are similar, but the sizes of MMEJ‐mediated deletions are somewhat larger.

**Figure 1:**
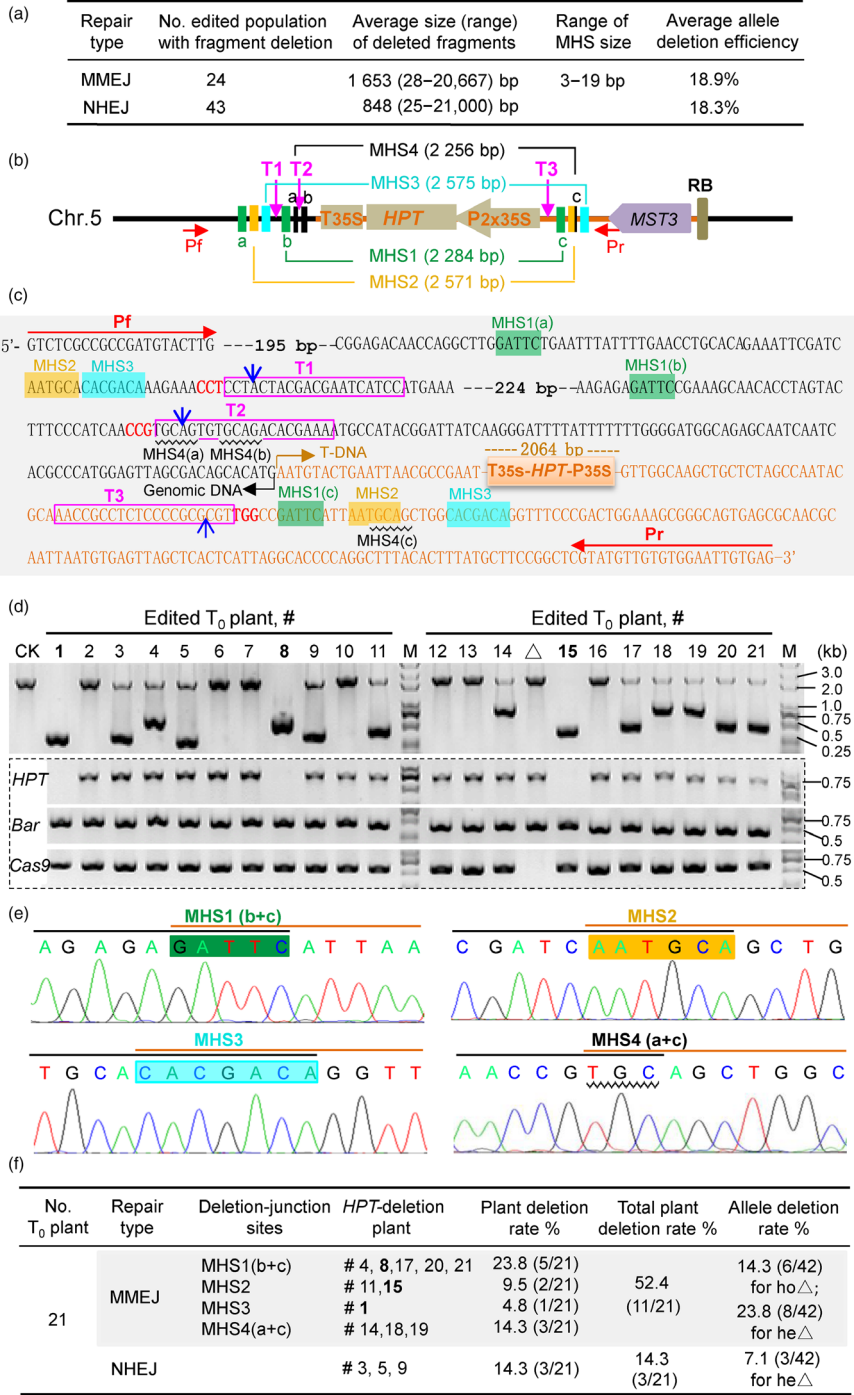
CRISPR/Cas9‐based genomic fragment deletion through MMEJ pathway. (a) Comparison of frequencies of genomic fragment deletions mediated by MMEJ and NHEJ in rice. An edited population stands a group of T_0_ individuals transformed with the same construct, which contains a proportion of MMEJ‐ or NHEJ‐mediated fragment deletion events or proportions of the both events. Forty‐eight such edited populations with fragment deletions were analysed, and 19 of them had both MMEJ‐ and NHEJ‐mediated fragment deletions. (b) Schematic diagram for the T‐DNA insertion in a *MST3*‐transgenic rice line, showing the designed target sites (T1–T3) and the MHSs (sites of the same group are shown in the same colour). (c) The sequences flanking the *HPT* cassette, indicating the MHSs and target sites, and the Cas9‐cutting positions (blue arrows). The Pf/Pr primer pair was used to detect fragment deletions. (d) Positive retransformed plants were confirmed by PCR to amplify the *HPT*, *Bar* and *Cas9* genes. A plant indicated with a triangle had a truncated T‐DNA with a deletion of *Cas9* (but retaining the *Bar* gene). PCR using the Pf/Pr primer pair detected the excision of the *HPT* cassette in a homozygous state (ho△, with one smaller band), a heterozygous state (he△, with two bands) and non‐deletion (with one large band). (e) Sequencing chromatograms showing four types of the deletion‐junction events in 11 MMEJ‐edited plants. Note that after cutting by Cas9 (c), MHS4 (a) had 3 bp in size. (f) A summary of the efficiencies of the marker gene deletion by MMEJ and NHEJ. The edited *HPT*‐excision plants by MMEJ (11 plants) and NHEJ (3 plants) are indicated.

We next attempted to induce MMEJ to delete a marker gene from an existing transgenic line. We previously generated a transgenic line carrying a homozygous, single‐copy T‐DNA insertion of a transgene carrying *MONOSACCHARIDE TRANSFERASE 3* (*MST3*). The *MST3* transgene was in a pCAMBIA1300‐based binary construct with the hygromycin‐resistance (*HPT*) gene cassette (Figure 1b). To delete this *HPT* cassette using genome editing, we first determined the sequences flanking the T‐DNA insertion by the modified high‐efficiency thermal asymmetric interlaced PCR (mhiTAIL‐PCR) (Tan *et al.*, 2019). We then used our online microhomology‐finder tool (http://skl.scau.edu.cn/repfinder/) to analyse the sequences flanking the *HPT* cassette in the T‐DNA and the inserted genome to identify MHSs. This analysis identified four groups of MHSs (MHS1–4) of 5–7 bp in the flanking sequences (Figure 1b,c). To produce DSBs as close as possible to the MHSs for efficient MMEJ‐mediated fragment deletion, we used the web‐based tool CRISPR‐GE (http://skl.scau.edu.cn/) to design three target sites (T1–T3) that were located near any pair of the MHSs, including two target sites on the genomic flanking sequence and one target site on the T‐DNA flanking sequence (Figure 1b,c).

To induce DSBs at the target sites, we prepared a binary construct with three single‐guide RNA (sgRNA) expression cassettes for the target sites based on the pYLCRISPR/Cas9P_ubi_‐B vector with the glyphosate selectable marker gene *Bar* as described (Ma *et al.*, 2015) and used it to retransform this transgenic line (Figure 1b,c). We obtained 21 *Cas9*‐positive T_0_ plants as confirmed by PCR (Figure 1d). PCR with a Pf/Pr primer pair (Figure 1b,c) detected 3 plants (#1, #8 and #15) with homozygous deletion (ho△) and 11 plants (#3–#5, #9, #11, #14 and #17–#21) with heterozygous deletion (he△) (Figure 1d).

To further determine whether these deletions were mediated by MMEJ or NHEJ, we examined the deletion‐junction sequences. To this end, all the PCR‐amplified fragments with reduced sizes and full length (Figure 1d) were recovered and sequenced. In the three ho△ plants, the *HPT* cassette was precisely excised with deletion‐junction events between the pair of MHS1, MHS2 or MHS3 (Figure 1e,f). Of the 11 he△ plants, 8 had deletion‐junction events between pair of MHS1, MHS 2 or MHS 4 (Figure 1f). The remaining 3 he△ plants (#3, #5 and #9) had fragment deletions generated by NHEJ, with junction sites between the targeted non‐homologous DSB ends. In these he△ plants, sequencing of the full‐length fragments showed the presence of the *HPT* cassette but with various base insertion/deletion mutations at the three targeted sites. In total, 3/21 (14.3%) of the edited plants had a homozygous marker gene deletion by MMEJ, and 11/21 (52.4%, including 8 plants by MMEJ and 3 plants by NHEJ) underwent mono‐allelic (heterozygous) gene deletion (Figure 1f). Our results demonstrate that by designing suitable target sites near MHSs (see below), the efficiency of MMEJ‐mediated fragment deletion is much higher than that of NHEJ‐mediated deletions. In addition, we obtained marker‐free T_1_ plants derived from the ho△ #1, #8 and #15 plants, which had no *HPT* and the T‐DNA (containing the CRISPR/Cas9 components and *Bar*) removed by genetic segregation.

Although studies have reported genomic fragment deletions by CRISPR/Cas systems, the efficiency is not high. The MMEJ pathway is active during cell cycle, which provides more chances for DSB repair than the other DNA repair pathways (Shen *et al.*, 2017). Our results suggest that three factors may be related to the efficiency of genomic deletion using MMEJ‐assisted CRISPR/Cas editing. First, the MHS1 and MHS4 sites, which are closer to the targeted sites (Figure 1c), had higher efficiency (in total 8/21) than that of the other two MHSs (in total 3/21, Figure 1f). It seems that the closer the MHSs are to the DSB positions, the higher the fragment deletion efficiency will be. Second, the length of MHSs may be less important than their positions relative to the DSBs; for example, MHS3 has the longest size (7 bp) but it produced only one he△ plant. Therefore, a MHS size of 4–5 bp (or even 3 bp, such as MHS4(a)) is sufficient for efficient MMEJ‐mediated DNA repair. Third, production of multiple DSBs using multiplex sgRNAs may increase the frequency of genomic fragment deletions.

In conclusion, the high‐efficiency genomic fragment deletion strategy using MMEJ‐assisted CRISPR/Cas9 editing is flexible in target design for many genomic regions to remove targeted genomic fragments. Moreover, this approach can facilitate functional genomics research and genetic improvement of organisms.

## Conflict of interest

The authors declare no conflict of interest.

## Author contributions

Y.‐G.L. and Q.Z. designed the studies. J.T., Y.Z., B.W., Y.H., Y.W., Y.L., W.L., W.Z., G.L., S.C., K.M. and X.X. performed the experiments. J.T., L.C., Q.Z. and Y.‐G.L. analysed data. Q.Z., Y.‐G.L. and J.T. wrote the paper.
